# Neuroplasticity at Home: Improving Home-Based Motor Learning Through Technological Solutions. A Review

**DOI:** 10.3389/fresc.2021.789165

**Published:** 2021-12-21

**Authors:** Christian Riis Forman, Jens Bo Nielsen, Jakob Lorentzen

**Affiliations:** ^1^Department of Neuroscience, University of Copenhagen, Copenhagen, Denmark; ^2^Elsass Foundation, Charlottenlund, Denmark; ^3^Department of Pediatric Neurology, University Hospital Rigshospitalet, Copenhagen, Denmark

**Keywords:** motor learning, digital rehabilitation, neurorehabilitation, telerehabilitation, neural plasticity

## Abstract

**Background:** Effective science-based motor rehabilitation requires high volume of individualized, intense physical training, which can be difficult to achieve exclusively through physical 1-on-1 sessions with a therapist. Home-based training, enhanced by technological solutions, could be a tool to help facilitate the important factors for neuroplastic motor improvements.

**Objectives:** This review aimed to discover how the inclusion of modern information and communications technology in home-based training programs can promote key neuroplastic factors associated with motor learning in neurological disabilities and identify which challenges are still needed to overcome.

**Methods:** We conducted a thorough literature search on technological home-based training solutions and categorized the different fundamental approaches that were used. We then analyzed how these approaches can be used to promote certain key factors of neuroplasticity and which challenges still need to be solved or require external personalized input from a therapist.

**Conclusions:** The technological approaches to home-based training were divided into three categories: sensory stimuli training, digital exchange of information training, and telerehabilitation. Generally, some technologies could be characterized as easily applicable, which gave the opportunity to promote flexible scheduling and a larger overall training volume, but limited options for individualized variation and progression. Other technologies included individualization options through personalized feedback that might increase the training effect, but also increases the workload of the therapist. Further development of easily applicable and intelligent solutions, which can return precise feedback and individualized training suggestions, is needed to fully realize the potential of home-based training in motor learning activities.

## Introduction

Neurological disorders are the leading cause of physical disability in the world (1, 2), and the rehabilitation efforts can be highly demanding both to the individual (3) and to the healthcare system (4). Increased focus on prevention, care and rehabilitative activity will therefore be of great significance to reduce the future economic burden on the public healthcare systems.

Attention should be directed toward optimal motor learning based on current research, with the purpose of achieving the best functional result. Even though the functional outcome of the individual with a brain lesion is influenced by several factors, such as the location and size of the lesion (5), adaptations in the nervous system after the lesion goes on throughout our lives influenced by our genes and how these are exposed to the surroundings. The changes to the connections in the brain, that accompany learning and memory, imply physical changes in the neural network (6). We refer to this phenomenon as neural plasticity. Research into neurorehabilitation is now providing insights, making it possible to develop guidelines for effective neurorehabilitation based on optimizing neural plasticity, which can be tailored to the needs and possibilities of the individual (7–9). The many factors for achieving the optimal neurorehabilitation should all be considered when designing a training schedule for an individual with a neurological disorder, but some are more difficult to include than others are, during personal rehabilitation training in the clinic. For example, to induce positive neuroplastic changes there appears to be no “upper limit” to the amount of training that can be done, and training every day and even several times per day appears to be possible and to some extent beneficial. It is however not an easy task for neither the therapist nor the individual to meet for rehabilitation training so frequently. Therefore, to perform such a high amount of training, it is necessary to prescribe training performed at home. The basic form of home-based training for neurorehabilitation purposes is the training program given by the therapist either verbally or in writing, which the individual or caretaker brings home and performs regularly on their own accord. As information- and communications technology is increasingly integrated into the everyday lives of many people, new opportunities have emerged to facilitate home-based training. A simple addition to a home-based training program could be a sensory stimulus e.g., an auditory stimulus to facilitate a certain rhythm or a visual stimulus to ensure correct training performance or motivation. A further level of technology to add to the home-based training is to include interactivity between a sensory stimulus and the actions of the individual. This type of interaction is the basis of video gaming and is therefore achievable by modifying video games into exercise sessions through special sensors or controllers, also sometimes referred to as exergaming (10). Whereas, these first two options merely provide interaction between the active individual and a technological device, another option is to facilitate interaction from home with the therapist in the clinic through communications technology. This method is most commonly referred to as telerehabilitation (11). In this article, we aimed to review the many different ways these information- and communications technology can be used to create the optimal setting for home-based neurorehabilitation training for both children and adults with neurological disorders, and to compare them to the general principles of neuroplasticity to discuss their strengths and uses.

## Litterature Search

A systematic literature search was performed to identify original research studies through the databases PubMed/Medline, CINAHL, PEDro, and Embase according to the PICO criteria. Both MeSH terms and keywords (sometimes truncated) were used in different combinations. The words used in Population was: “Neurological disorders,” “Cerebral palsy,” “Stroke,” “Multiple sclerosis,” “Spinal cord injury,” “Traumatic brain injury.” The words used in Intervention was: “home-based (training/rehabilitation/exercise/activity/physiotherapy),” “video games,” “exergaming,” “telerehabilitation.” Comparator and outcome were not included in the search string, but instead used in the following screening. We selected studies where neurorehabilitation training was being performed in the home setting, facilitated by information- and communications technology. We were only interested in interventions performed outside of the clinic without the physical presence of a trainer. This meant rejecting studies where the majority of the training was carried out during home visits by a trainer/therapist. Home visits by trainers for introduction or adjustments of the training program were accepted. It also meant rejecting home-applicable training (e.g., using commercial video game consoles) where the project was performed in a clinic. For comparator group we only included studies, which contained a control group. The control group could be receiving a clinic-based intervention, a different home-based intervention or no intervention at all. The screening was performed by two independent researchers on first abstract/title and subsequently on the full text. Any disagreements were afterwards discussed case-by-case. The literature search identified 634 unique original publications, which were to be screened for eligibility according to this study's criteria. Four hundred twenty-five studies were excluded from the screening of title/abstract, which left 209 studies to assess from full text. A further 181 studies were excluded from the full text assessment, leaving 28 studies to be included. The most common reasons for study exclusion was lack of technological solutions, performance of the study in a laboratory setting or constant physical presence of a trainer. We then grouped the included studies into the following three categories: (1) “Sensory stimuli training,” defined as neurorehabilitation training where the training individual received live sensory input, but no live information was returned. (2) “Digital exchange of information training” where interactivity between sensory stimuli and the recorded actions of the individual was able to personalize the training course independent of input from the trainer. (3) “Telerehabilitation,” where the individual and trainer can communicate directly through the use of technology, closely mimicking the conditions of a clinical visit but without the physical presence.

## Sensory Stimuli Training

Home-based training provides the opportunity to be less dependent on the schedule and location of the therapist, and instead perform the training at the individual's optimal time of the day, which gives the chance to prescribe a higher training volume. One way to improve home-based training interventions includes technological inputs that involve sensory stimuli using simple and easy-to-use technology such as videos, audio-players or audio metronomes. Other interventions provide elaborate enriched environments of sensory stimuli tailored to the individual (12). These types of training promote easier goal setting in the home-based training by sensory stimuli that motivates the individual to perform at a certain tempo or to complete a certain set of exercises in a particular order. The active participation in the training is limited to following the sensory stimuli without influencing them, but the simplicity of use provides opportunity for many repetitions, though in many cases few possibilities for variation in training. One way to provide variation is to include a wide selection of different exercises. These can then be individualized (12–14), but the potential for variation is limited and requires additional time and effort from the trainer in order to prescribe the correct selection. There is a similar difficulty in providing progressive challenges. Rhythmic auditory stimulation walking programs (15–17) can provide progression through scheduled increases of tempo, but visual stimulus training programs will require personal trainer assessments to find a proper next level of challenge. Some studies have tried to provide challenges to sensory stimuli programs by giving additional training equipment that increases difficulty, e.g., blindfolds, incline surfaces or foam pads for balance training (18, 19) or weighted vests for resistance training (19, 20). Sensory stimuli training can also be applied in infants aged 3–6 months with increased risk of developing cerebral palsy (CP) due to premature birth (12, 14). Here, an interactive enriched environment, which was designed specifically to the infants' needs showed the possibility of providing training at home for even very small infants. This method involved remote individually selected exercises, goal-setting, variation, and feedback through interaction with a therapist. Simple modifications of sensory feedback during the training tasks can be an efficient way of providing variation and challenge to the training of walking, balance or similar motor tasks. Since no exchange of information is made between the sensory stimulus technology and the performance of the training, the immediate feedback and rewards are limited to the individual's own personal assessment of the performance. With rhythmic auditory stimulation training (15–17, 21), it is relatively easy to self-assess the step frequency compared to the auditory stimulus, but with exercise videos, it can be more difficult to know whether the exercise is being performed well. In these cases, regular assessment of the exercise performance from the trainer becomes important for reducing injuries and providing the feedback of successful improvements. Sensory stimuli training therefore seems to be of most use when the exercise task is relatively simple and benefits from a high amount of repetitions without significant variations and when the individual has a high level of internal motivation or is motivated by a caregiver.

## Digital Exchange of Information Training

Active participation during the practice of the tasks is necessary if the aim is to induce neuroplastic changes and promote learning and function (22–24). To continue inducing neuroplastic changes, the task level of difficulty must also be progressively increased over time (25, 26). Furthermore, the training should be motivating to the individual and should include clear goal setting, feedback on the performance and rewards for desired achievements to improve the learning outcome. Digital exchange of information training includes technology that provides various sensory stimuli in a similar way to the sensory stimuli training but differs by also being able to record input from the active individual through sensors or controllers. The increased use of technology slightly increases the requirements of the right time and place for a training session compared to simple sensory stimuli training, but in most cases, this training form still maintains enough simplicity to promote many repetitions and a high training frequency. Goal-setting is relatively easy as most games or exercises will have a simple and easy to understand task to be completed, but the feedback given is higher than in sensory stimuli training, as the computer systems themselves are able to provide some measure of performance, often in the form of a performance score. From this performance score, the system can give a progressively harder challenge or a different task variation. One study developed an automated evaluation of motor function that was integrated into the digital training system (27), and which helped to evaluate the current requirements of the individual during the specific point of the program. Such an addition to the training program can be efficient in providing both variation and progressive challenges without being time-consuming for the trainer. However, in the commercial or widely available software systems, a score could risk not being specific enough to the actual quality of the movements performed and in these cases, frequent feedback from the trainer would be required to ensure proper exercise performance, progression and variation. Furthermore, the selection of training tasks will often be limited by the data input through the sensors or controllers, and complex movement tasks can therefore be a challenge to include. Several projects have attempted to improve the training options by creating their own customized setups (27–34). Some studies have made customized sensors to include more complex movements such as precise hand and finger positions (27, 33), whereas others have swapped video gaming controllers for video recordings of dexterity tasks with physical tools (30) or goniometers for tracking tasks (28, 29). The customized tasks allow for better targeting the challenges of the individual group of neurological disorders and perhaps fine-tuning the progression of the training task more precisely. It is a strength of the digital exchange of information training, that data from the performance of the training is available digitally for both instant or delayed evaluation and feedback from the trainer. This feature has been integrated into some training programs in different ways. Two studies give an example of delayed feedback once/week (31, 35), where trainers provided feedback and training adjustments through digital communication based on the individual's performance data from the previous week. Though this feature promotes neuroplasticity through feedback, progressive challenges and variation, it requires some effort from the trainer. Developing the feedback options further, other studies have integrated digital instant feedback from the trainer and achieved a setup with communication similar to this study's definition of telerehabilitation (28, 29, 36, 37). Overall, training programs involving digital exchange of information might facilitate neuroplasticity in home-based training by giving feedback, either automated or delayed by checking performance data, by increasing motivation and repetitions of perhaps otherwise trivial activities, and by giving easily-understandable, although limited, task variations, or challenges.

## Telerehabilitation

It can be beneficial to improve motor function in familiar and everyday-applicable surroundings and cost-efficient both for the individual not requiring transportation and for the healthcare system if it can rely more on digital automated training delivery and less on physical 1-on-1 consultations. Home-based training provides new opportunities, but it also gives different challenges to solve. The lack of direct physical supervision challenges the training planning in terms of being certain that the training accomplishes optimal rehabilitation results. These include ensuring active participation during the training, providing the appropriate level of progressive challenge and variation, and giving precise feedback at the right time, which are all difficult without the trained eye of an experienced therapist. To increase the potential of home-based training, it is therefore important to consider how to promote the implementation of these principles of neuroplasticity in the home-based training program. Telerehabilitation is an emerging field within health and rehabilitation, and many definitions exist on what defines it. Videoconferencing is a common method and likely the method, which most closely mimics the conditions of a physical session in both strengths and weaknesses. The live attention to the individual makes it possible for the trainer to tailor the progression and variation in exercises to the individual's needs, to continuously set individual goals and give feedback on their achievement, and to facilitate the active participation in the completion of different tasks. One challenge to providing the right exercise variations or progressive challenges, however, is the selection of available equipment or usable objects in the home. One study attempted to improve the task options by providing simple manual objects and tasks (i.e., toys) to promote manual dexterity training (38). This could be a simple and cheap way for the trainer to motivate and vary the tasks. The challenges of telerehabilitation through videoconferencing is that it requires more time and resources for the trainer to be available live. It is therefore a challenge to achieve the many repetitions necessary to achieve the optimal stimulus for neuroplasticity. Two studies using videoconference managed to provide supervised individual telerehabilitation 5 times/week (39, 40), which is an impressive feat, but, would likely not be feasible in many rehabilitation clinics. Another study attempted to combine telerehabilitation with a traditional home-based training program (38) to achieve a higher overall amount of repetitions. Whatever the method, aiming to increase the amount of repetitions performed seems like the important focus when planning telerehabilitation through videoconferencing. The other telerehabilitation method, e-training platforms, where the communication is through asynchronous text messaging has another set of possibilities and challenges. The training is not dependent on the trainer being available and so does not limit the potential for many repetitions. Furthermore, since the trainer can send both detailed descriptions and pictures or videos of suggested exercises this method has more potential for suggesting variations or progressive challenges than a traditional home-based training program. The feedback is given delayed and based on written comments/diary entries (41–43) or by a simple exertion score (44). This limits the level of detail that the trainer can use for the feedback, which is especially a challenge if the task is a complex motor task. The two types of telerehabilitation included in this category are therefore quite different from each other. Deciding which type would be most efficient for a training program requires an analysis of the specific goal and whether this goal benefits more from many repetitions or precise feedback and individual progression. For some training programs, a mix of the two might be the optimal method. The ratio of supervised videoconference training to unsupervised e-training prescribed training would depend on the demands of the specific exercises and the ability level of the exercising individual. This is likely what many studies across categories have aimed at by providing regular videoconference conversations for feedback. Although this undoubtedly facilitates motivation, the lack of live performance of exercises might make the trainer miss important observations on which to progress or vary the training, or give the proper feedback.

## What is the Role of the Therapist?

The main role of the therapist in relation to rehabilitation of individuals with disabilities due to neurological disorders is providing training that drives neuroplastic and functional changes in the optimal way. The continuous optimization of the training, which is important to make the best progression, currently requires involvement of the therapist. Clear goals accompanied by rewards are indispensable in this situation, but difficult to administer if the therapist is not present during the actual training. It is necessary to find methods that combine (1) high dose of training with (2) influence of the therapist ensuring the optimal performance of the training and motivation (3) time- and cost efficient for both the individual and rehabilitation system. The use of information and communications technology in home-based training is seemingly able to promote the different factors that are important for inducing neuroplastic changes, but each modality comes with its own set of opportunities and challenges. Generally, the training technologies are stuck in a trade-off between high dose/many repetitions and a high degree of therapist feedback and input unless they are highly resourceful in terms of providing personal time and attention. Digital automated feedback can be part of the solution and comes in the form of simple performance scores after an exergaming task or more complex movement analyses using movement sensors and trackers. Training programs involving specific tasks, requiring a low amount of motor skill and benefitting from many repetitions, can already make use of these information- and communications technologies, whereas more technological development might be needed to give the proper feedback in more complex motor tasks. The role of the therapist in this situation is to analyze the needs of the specific population and training tasks and prescribe the optimal training schedule and technological methods, keeping in mind that the best training outcome might results from a combination of different technologies. Also, until the use of information- and communications technology is developed further for use in training, many training programs will still require frequent personal input from the therapist in order to provide feedback, optimize training performance and motivate. Motivation is a critical element of rehabilitation to remember (45) and is identified by some physiotherapists as the single most influential personal characteristic that determines motor and functional outcomes in physiotherapy (46). One of the biggest challenges in any training plan is to maintain the motivation over long periods necessary to achieve significant and lasting functional improvements. This is even more challenging when training has to be performed at home alone in order to achieve the necessary frequency and intensity of training. As motivation can be a highly personal factor compared to the other factors for neuroplasticity, it is important that the therapist considers the individuals personal motivation when designing a home-based training plan. Where some individuals might find enjoyment and motivation in the gamification of a task, others might consider it confusing and unnecessary and thereby lose motivation. Knowing what motivates a specific person is still something that requires personal interaction and empathy from the therapist. Overall, though information- and communications technology has given many new opportunities to integrate neurorehabilitation training into the home-based setting, there are still several important roles for the therapist to play.

## Conclusion

Home-based training, with the addition of technological solutions, can be of notable value to motor learning and neurorehabilitation by promoting various factors of neuroplasticity. Generally, home-based training can promote a larger training volume and more flexible scheduling according to the needs of the individual. With the addition of technology, important neuroplastic factors such as goal-setting, feedback, rewards, and motivation can be stimulated, enabling better results. Other factors of neuroplasticity can be slightly more challenging to include in home-based training such as progressive challenges, variation and ensuring the highest level of active participation. Some technological training solutions have attempted to solve this by including enriched physical environments or automated progression, but most often, the trainer is required to ensure the optimal inclusion of these factors, making it more expensive and labor-intensive. The general trade-off in the different technological solutions to home-based training therefore seems to be between more flexibility/training volume and optimal progression/variation/feedback. For that reason, identifying the most important factors of the specific motor learning program is key to implementing home-based training.

## Author Contributions

CF wrote the first draft of the manuscript. CF, JN, and JL contributed to the design of the project, critically revised the manuscript, and approved the final version. All authors contributed to the article and approved the submitted version.

## Funding

This study was funded by a personal grant from the Elsass Foundation to Professor Jens Bo Nielsen (Grant number 19-3-1081).

## Conflict of Interest

The authors declare that the research was conducted in the absence of any commercial or financial relationships that could be construed as a potential conflict of interest.

## Publisher's Note

All claims expressed in this article are solely those of the authors and do not necessarily represent those of their affiliated organizations, or those of the publisher, the editors and the reviewers. Any product that may be evaluated in this article, or claim that may be made by its manufacturer, is not guaranteed or endorsed by the publisher.
