# Supplementary material for: Neuroplasticity at Home: Improving Home-Based Motor Learning Through Technological Solutions. A Review
Source: Front Rehabil Sci. 2021 Dec 21;2:789165. doi: 10.3389/fresc.2021.789165 (PMC9397835; doi:10.3389/fresc.2021.789165)
Supplement: Supplementary file 1 [file Table_1.DOCX]

| **Category** | **Pros** | **Cons** | **Best used when** | **Beneficial additions** | **Exemplified by** |
| --- | --- | --- | --- | --- | --- |
| **Sensory stimuli**  **training** | Easy-to-use setup    High training volume/many repetitions  Low cost | Lack of personalization, variation and progression.  Requires a high degree of internal motivation. | Task is simple and benefits from being performed many times per week. | Enriching the environment to provide variation and progression | PDSAFE intervention [19] for stepping and fall-prevention. Videos of ideal and personal exercise technique were provided, high training volume many times/week was achieved, and variation and individualization was added using simple equipment. |
| **Digital exchange of information training** | Provides some performance feedback  Facilitate active participation  A stimulating software interface increases motivation  Provides easy goal-setting | Limited exercise selection and complexity.  Requires appropriate existing technology or labor-intensive development of new technology | External motivation is needed for achieving many repetitions.  Meaningful progression can be achieved by making small adjustments to the system. | Using performance data to provide personalized feedback and suggestions for progression | MusicGlove intervention [34] for hand motor rehabilitation. A custom built musical video game helped to facilitate active participation in hand grip training and provided some performance feedback through glove sensors. |
| **Tele-rehabilitation** | Provides individualized progressive challenges and variation.  Provides detailed performance feedback | Time-consuming for the trainer.  Low frequency and volume of training. | The task is complex or if incorrect performance can lead to detrimental consequences | Adding simpler forms of home-based training to increase amount of repetitions performed of select exercises | MS HAT system [42] included individualized exercise prescription, educational material and communication from the trainer to mimic a therapy session, however asynchronously and remote. |
